# Harnessing Twitter data to survey public attention and attitudes towards COVID-19 vaccines in the UK

**DOI:** 10.1038/s41598-021-02710-4

**Published:** 2021-12-14

**Authors:** Seena Fazel, Le Zhang, Babak Javid, Isabell Brikell, Zheng Chang

**Affiliations:** 1 Warneford Hospital, Department of Psychiatry, University of Oxford, Oxford, UK; 2 Department of Medical Epidemiology and Biostatistics, Karolinska Institutet, Stockholm, Sweden; 3 Division of Experimental Medicine, University of California San Francisco, San Francisco, USA; 4 National Centre for Register‐based Research, Department of Economics and Business Economics, Aarhus BSS, Aarhus University, Aarhus, Denmark

**Keywords:** Infectious diseases, Health policy

## Abstract

Attitudes to COVID-19 vaccination vary considerably within and between countries. Although the contribution of socio-demographic factors to these attitudes has been studied, the role of social media and how it interacts with news about vaccine development and efficacy is uncertain. We examined around 2 million tweets from 522,893 persons in the UK from November 2020 to January 2021 to evaluate links between Twitter content about vaccines and major scientific news announcements about vaccines. The proportion of tweets with negative vaccine content varied, with reductions of 20–24% on the same day as major news announcement. However, the proportion of negative tweets reverted back to an average of around 40% within a few days. Engagement rates were higher for negative tweets. Public health messaging could consider the dynamics of Twitter-related traffic and the potential contribution of more targeted social media campaigns to address vaccine hesitancy.

## Introduction

Several COVID-19 vaccines have been approved globally and broad scale vaccination is currently underway. However, attitudes towards vaccination and in particular what has been termed vaccine hesitancy, present a potential threat to achieving coverage and community immunity. A survey across 19 countries (collected June 16 to June 20, 2020, from an online panel of 13,426 respondents) found that 72% of participants were very or somewhat likely to take a COVID-19 vaccine, but with wide variations in acceptance rates between countries (e.g. from 90% in China to less than 55% in Russia)^1^. Older age, higher socioeconomic status, and trust in their government were associated with higher vaccine acceptance^1^. Similar results were found in a recent survey of UK adults, where 72% of participants reported willingness to be vaccinated whilst the remaining 28% were “very unsure” or “strongly hesitant”^2^. Attitudes about the collective importance, efficacy, side-effects, and speed of development of a COVID-19 vaccine were the most important variables predicting vaccine hesitancy, whilst sociodemographic factors (e.g. age, sex, and socioeconomic status) only explained 10% of variance^2^.

Vaccine hesitancy is not a new phenomenon, but as the use of social media platforms grows globally, barriers to the spread of anti-vaccination attitudes and misinformation have been removed. This is of potential concern, as social media has taken a significant role in the public discourse about COVID-19, with the World Health Organization (WHO) warning of an online ‘infodemic’ relating to COVID-19^3^. Prior research indicates that individuals who rely on social media for information regarding the pandemic are more likely to be vaccine hesitant^3,4^, and that anti-vaccine content is prevalent across social media platforms, posted by a minority of users, but frequently generates greater user engagement than neutral or pro-vaccine social media posts^5–7^. Moreover, recent studies found that social media posts with COVID-19 related misinformation are shared as often as those with information deemed reliable^8,9^, and that social media disinformation campaigns are associated both with drops in vaccination coverage (measured via annual data of actual vaccination rates from the WHO) and increased levels of negative vaccine discourse on Twitter^10^.

Vaccination programs have been moving from elderly and other high-risk groups to young and middle-aged adults and adolescents in many countries. As these younger age groups are those who most frequently use social media, real-time social media data can provide important information about trends in public attitudes and sentiment in response to news reports and public health interventions encouraging vaccine uptake^11^. Social media has been widely discussed as a potential platform for health communication as well as a tool for exploring health-related issues^12,13^, and several studies have examined the social media attention and attitudes towards COVID-19 vaccines in different countries^14–18^. A recent published work utilized topic modelling methods to identify topics related to COVID-19 vaccines on Twitter; the authors also calculated weekly sentiment^19^ but how public attitues interact with news about vaccine development and efficacy were not studied. In this study, we examined social media attention and attitudes to COVID-19 vaccine using Twitter posts in the UK.

## Results

### Change of Twitter volume over time

**Figure 1:**
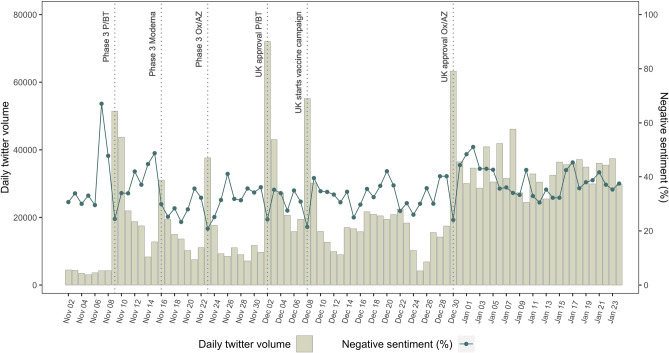
Social media attention to Covid-19 vaccine in UK from November 2020 to January 2021.

We obtained 1,929,473 original COVID-19 vaccine-related tweets from 522,893 unique users in the UK during November 2, 2020 to January 24, 2021. The range of daily COVID-19 vaccine-related tweets was 3049–72,027 (Fig. 1). In November 2020, COVID-19 vaccine-related tweets increased substantially with the release of trial results of the Pfizer/BioNTech, Moderna, and Oxford/AstraZeneca vaccines, then the volume decreased with time. Twitter volumes peaked with the approval of the first COVID-19 vaccine from Pfizer/BioNTech at 72,027 tweets, and dropped to a low level during the Christmas holiday period (mean daily volume of tweets 9201). After the approval of the Oxford/AstraZeneca vaccine, the Twitter volumes increased again and remained at a high level with the phase one rollout of vaccination continued (mean daily volume of tweets 33,686).

### Public attitudes towards COVID-19 vaccines

The percentage of negative sentiment tweets varied from 20.7% to 51.1% (excluding the reference week). The percentage of negative sentiment dropped with every new announcement (e.g., 24.4% with Phase 3 trial results from Pfizer/BioNTech, 20.9% with Phase 3 trial results from Oxford/AstraZeneca, 21.5% with UK starting its vaccine campaign), but then reverted to a higher level (36.4%) after each news announcement.

**Figure 2:**
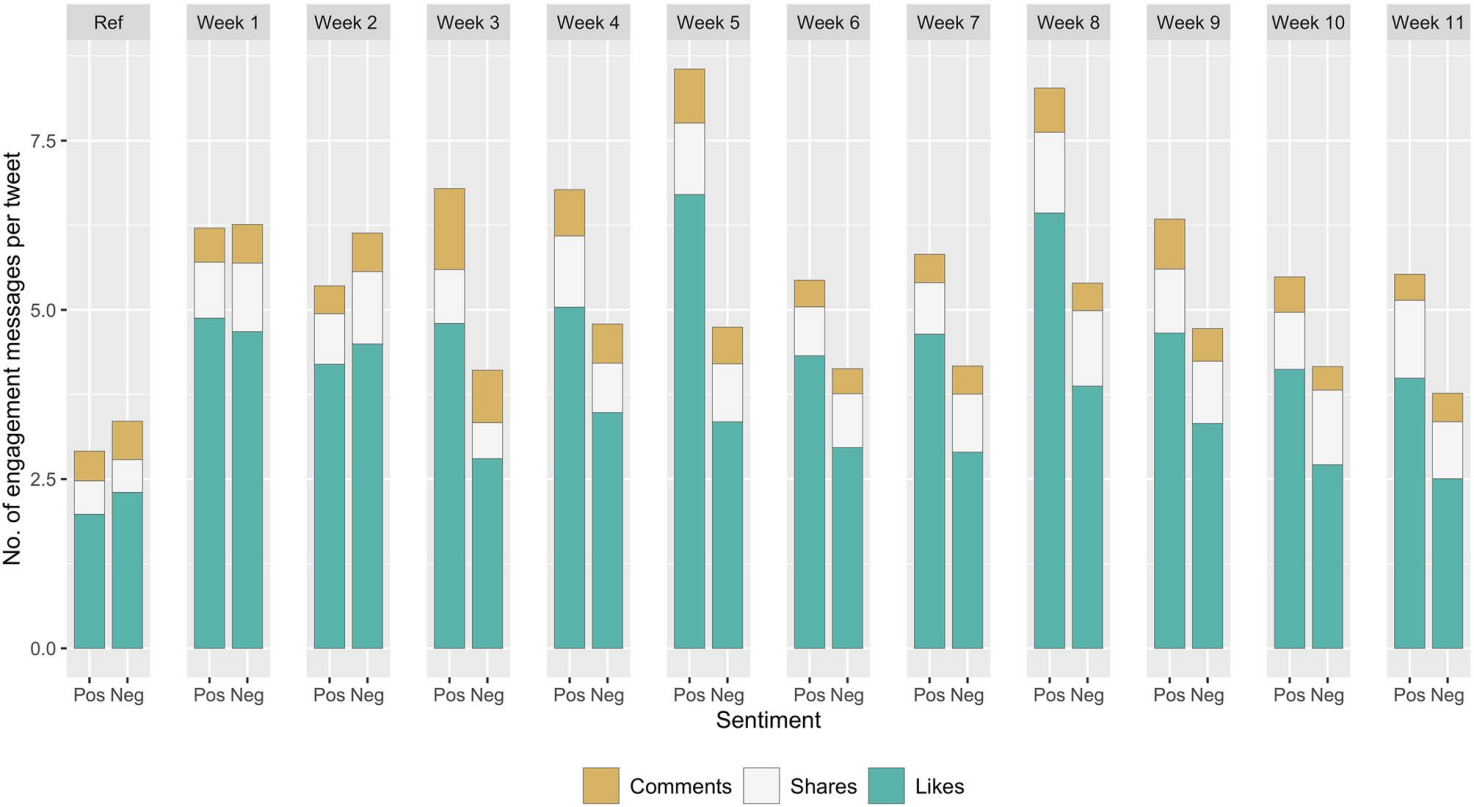
Average number of engagement for positive and negative tweets from November 2020 to January 2021, by week.

Negative tweets were posted by a smaller number of unique individuals during the study period, compared to tweets presenting positive views (38 vs 45 unique authors per 100 tweets). The engagement pattern for positive and negative tweets changed over time (Fig. 2). When the first trial results were released in November, negative tweets had higher engagement rates than positive tweets, but negative engagnment rates declined after the release of Oxford/AstraZeneca Phase 3 results (week 3). This was primarily driven by the decreasing number of likes for negative tweets (mean number of likes for negative tweets: 4.6 in week 1 and 2 vs. 3.1 in week 3–11, whereas mean number of likes for positive tweets increased: 4.5 in week 1 and 2 vs. 5.0 in week 3–11).

## Discussion

Using Twitter data from the UK during November 2020 to January 2021, we investigated the ecological associations between vaccine-related major news announcements, and attitudes towards vaccines. This period coincided with news on the major vaccine trials being announced or published, and approvals by the UK Medicine and Health Regulations Authority (MHRA).

We report two main findings. First, each major news announcement related to vaccines was associated with a large decrease in negative sentiment on the same day, dropping from around 40% to 20% of all daily tweets. However, this was short-lived, and the proportion of negative tweets reverted back to the background average within a few days. A similar pattern of decreasing in negative sentiment when Pfizer/BioNTech announced its phase III vaccine trial results has been found^19^. This study also found a fluctuation pattern of public sentiment during the same period but other major news announcements were not investigated. Another study analyzing UK public sentiments toward COVID-19 vaccines on Twitter and Facebook also found public sentiment was potentially associated with news on vaccine development, although their study period ended in November 2020^15^ (whereas our study period started in November 2020). Second, tweets with negative sentiment towards vaccines were posted by a smaller number of unique individuals, compared to tweets presenting positive views. Negative tweets were more likely to be liked and retweeted when the trial results were initially released in November, but their popularity gradually decreased with the vaccination campaign underway.

Our data are limited by not having more information on the demographic factors associated with the tweets and the algorithm, which is necessarily limited by the nature of Twitter’s free text interface. In addition, we examined the absolute change in number regarding COVID vaccine-related tweets during the study period, but were not able to examine the trend relative to the total Twitter traffic, as the background volume of tweets on the platform was not available. This is consistent with previous work using social media to examine health-related content over time^20,21^. Furthermore, we did not take bot messages into consideration when including COVID-19 related tweets. However, as we examined the trend in number of tweets and engagement of tweets by day/week, this might not bias the results if the publishing of bot messages is non-differential by time. Finally, this investigation is based in one country, and only harvests information in English, despite many languages being spoken in the UK and possible differential attitudes across them.

Our results can inform public campaigns aimed at promoting vaccine take-up. They suggest that information campaigns need to be sustained beyond major news announcements. In addition, public education could consider the dynamics of the Twitter-related traffic on this issue by spacing out news announcements and repeating news stories about the vaccination programme, beyond simply publishing vaccination numbers. One possibility is regular news releases, specifically allied to tweets and other social media posts with more scientific content, as one way to develop a more informed discourse in the public sphere. This view is supported by research on attitudes before vaccines were available in Israel, which found an overall vaccine hesitancy rate of 25%^22^, whereas Israel has subsequently achieved very high rates of vaccine take-up^23^. This suggests that attitudes to vaccination may, in a proportion of people, be malleable and permeable to public health messaging.

## Methods

To study social media attention and attitudes to COVID-19 vaccine news announcement, we obtained COVID-19 vaccine related tweets in the UK from November 2, 2020 to January 24, 2021 via Sprout Social—Twitter official partner platform^24^. Tweets were collected using following keywords: covid, covid19, covid-19, covid_19, coronavirus, corona virus, covid19uk, vaccine, vaccines, vaccination, vaccinate, vaxx, oxford, astrazeneca, oxford/astrazeneca, oxford-astrazeneca, pfizer, biontech, pfizer/biontech, pfizer-biontech, moderna. Detailed search strategy is described in Appendix 1.

We plotted the number of vaccine related tweets by day and indicated when major news were announced using RStudio Version 1.1.463. During the study period, major news releases about COVID-19 vaccine include announcements of trial results, and authorisation and distribution of COVID-19 vaccine from major manufacturers (i.e., Pfizer/BioNTech, Moderna, and Oxford/AstraZeneca) in the UK.

Using a hybrid algorithm combining machine learning and rule-based approaches, each tweet was classified as expressing positive, negative, or neutral sentiment. Several steps of pre-processing including part-of-speech tagging, lemmatization, prior polarity, negations, amplifiers & other grammatical constructs were done before the machine learning model was performed. The machine learning model used by Sprout Social was built on a dataset of 50,000 tweets drawn randomly from Twitter. 10,000 tweets were used to test and tune the algorithm, none of which were used for building the algorithm. As the tweets are not specific on domains, the sentiment analysis could be performed on a wide range of domains^25^. Combining machine learning and rule-based approaches have been widely used to estimate the public sentiment^26,27^, and such methods have been shown to improve the effectiveness of sentiment analysis^25,27^.

The daily proportion (%) of negative tweets among the COVID-19 vaccine-related tweets dataset was calculated during the study period. Engagement with COVID-19 vaccine-related tweets was measured as average number of comments, shares and likes of the original tweet. We compared the average number of engagements with negative and positive tweets and present the figures week by week (reference week: Nov 2 to Nov 8, 2020; week 1: Nov 9 to Nov 15, 2020; week 2: Nov 16 to Nov 22, 2020 … week 11: Jan 18 to Jan 24, 2021).

## Supplementary Information

Supplementary Information.

## References

[1] Lazarus JV (2020). A global survey of potential acceptance of a COVID-19 vaccine. Nat. Med..

[2] Freeman D (2020). COVID-19 vaccine hesitancy in the UK: The Oxford Coronavirus Explanations, Attitudes, and Narratives Survey (OCEANS) II. Psychol. Med..

[3] Burki T (2020). The online anti-vaccine movement in the age of COVID-19. Lancet Digit. Health.

[4] Jennings W (2021). Lack of trust and social media echo chambers predict COVID-19 vaccine hesitancy. medRxiv.

[5] Puri N, Coomes EA, Haghbayan H, Gunaratne K (2020). Social media and vaccine hesitancy: New updates for the era of COVID-19 and globalized infectious diseases. Hum. Vaccin. Immunother..

[6] Blankenship EB (2018). Sentiment, contents, and retweets: A study of two vaccine-related twitter datasets. Permanente J..

[7] Johnson NF (2020). The online competition between pro- and anti-vaccination views. Nature.

[8] Cinelli, M. *et al.**The covid-19 social media infodemic*. arXiv preprint, arXiv:2003.05004 (2020). 10.1038/s41598-020-73510-5 PMC7538912 33024152

[9] Kouzy R (2020). Coronavirus goes viral: Quantifying the COVID-19 misinformation epidemic on Twitter. Cureus.

[10] Wilson SL, Wiysonge C (2020). Social media and vaccine hesitancy. BMJ Glob. Health.

[11] Merchant RM, South EC, Lurie N (2021). Public health messaging in an era of social media. JAMA.

[12] Sharma M, Yadav K, Yadav N, Ferdinand KC (2017). Zika virus pandemic—Analysis of Facebook as a social media health information platform. Am. J. Infect. Control.

[13] Moorhead SA (2013). A new dimension of health care: Systematic review of the uses, benefits, and limitations of social media for health communication. J. Med. Internet Res..

[14] Bonnevie E, Gallegos-Jeffrey A, Goldbarg J, Byrd B, Smyser J (2021). Quantifying the rise of vaccine opposition on Twitter during the COVID-19 pandemic. J. Commun. Healthc..

[15] Hussain A (2021). Artificial intelligence-enabled analysis of public attitudes on Facebook and Twitter toward covid-19 vaccines in the United Kingdom and the United States: Observational study. J. Med. Internet Res..

[16] Marcec R, Likic R (2021). Using Twitter for sentiment analysis towards AstraZeneca/Oxford, Pfizer/BioNTech and Moderna COVID-19 vaccines. Postgrad. Med. J..

[17] Cotfas L-A, Delcea C, Gherai R (2021). COVID-19 vaccine hesitancy in the month following the start of the vaccination process. Int. J. Environ. Res. Public Health.

[18] Kwok SWH, Vadde SK, Wang G (2021). Tweet topics and sentiments relating to COVID-19 vaccination among Australian Twitter users: Machine learning analysis. J. Med. Internet Res..

[19] Lyu JC, Le Han E, Luli GK (2021). COVID-19 vaccine-related discussion on Twitter: Topic modeling and sentiment analysis. J. Med. Internet Res..

[20] van Lent LG, Sungur H, Kunneman FA, van de Velde B, Das E (2017). Too far to care? Measuring public attention and fear for ebola using Twitter. J. Med. Internet Res..

[21] McClellan C, Ali MM, Mutter R, Kroutil L, Landwehr J (2016). Using social media to monitor mental health discussions—Evidence from Twitter. J. Am. Med. Inform. Assoc..

[22] Dror, A. A. *et al. Vaccine hesitancy: the next challenge in the fight against COVID-19*, 10.1007/s10654-020-00671-y (2020). 10.1007/s10654-020-00671-y PMC8851308 32785815

[23] Ritchie, H. *et al. Coronavirus (COVID-19) Vaccinations. Our World in Data*, https://ourworldindata.org/covid-vaccinations (2021).

[24] *Sprout Social - Twitter Official Partners*, https://partners.twitter.com/en/partners/sprout-social (2021).

[25] Boyce, J. *Sentiment Analysis 101: How Sprout’s Data Science Team Built a Hybrid Model*, https://sproutsocial.com/insights/sentiment-analysis/ (2017).

[26] Ribeiro FN, Araújo M, Gonçalves P, Gonçalves MA, Benevenuto F (2016). Sentibench-a benchmark comparison of state-of-the-practice sentiment analysis methods. EPJ Data Sci..

[27] Prabowo R, Thelwall M (2009). Sentiment analysis: A combined approach. J. Informetr..

