# Supplementary material for: Harnessing Twitter data to survey public attention and attitudes towards COVID-19 vaccines in the UK
Source: Sci Rep. 2021 Dec 14;11:23402. doi: 10.1038/s41598-021-02710-4 (PMC8671421; doi:10.1038/s41598-021-02710-4)
Supplement: Supplementary file 1 — Supplementary Information. [file 41598_2021_2710_MOESM1_ESM.docx]

Appendix

Search strategy for capturing COVID-19 vaccines related tweets in UK across the period Nov 2, 2020 to Jan 24, 2021.

COVID-19 vaccines related tweets were identified by the following search strategy consisting of case-insensitive hashtags (indicated by the symbol "#") and search terms:

("covid" OR "#covid" OR "covid19" OR "#covid19" OR "covid-19" OR "covid_19" OR "#covid_19" OR "coronavirus" OR "#coronavirus" OR "corona virus" OR "corona" OR "#corona" OR "virus" OR "#virus" OR "#covid19uk") AND ("vaccine" OR "#vaccine" OR "vaccines" OR "#vaccines" OR "vaccination" OR "#vaccination" OR "vaccinate" OR "vaxx") OR ("oxford" OR "astrazeneca" OR "oxford/astrazeneca" OR "oxford-astrazeneca" OR "pfizer" OR "biontech" OR "pfizer/biontech" OR "pfizer-biontech" OR "moderna") AND ("vaccine" OR "#vaccine" OR "vaccines" OR "#vaccines vaccination" OR "#vaccination" OR "vaccinate" OR "vaxx") OR "#covidvaccine" OR "#covid19vaccine"
